# Synthesis of *N*^4^-acetylated 3-methylcytidine phosphoramidites for RNA solid-phase synthesis

**DOI:** 10.1007/s00706-022-02896-x

**Published:** 2022-02-22

**Authors:** Sarah Moreno, Laurin Flemmich, Ronald Micura

**Affiliations:** Institute of Organic Chemistry, Center for Molecular Biosciences Innsbruck, University of Innsbruck, Innrain 80-82, 6020 Innsbruck, Austria

**Keywords:** Nucleosides, Nucleotides, Bioorganic chemistry, Solid-phase synthesis, RNA methylation

## Abstract

The growing interest in 3-methylcytidine (m^3^C) originates from the recent discoveries of m^3^C modified tRNAs in humans as well as its intensively debated occurrence in mRNA. Moreover, m^3^C formation can be catalyzed by RNA without the assistance of proteins as has been demonstrated for a naturally occurring riboswitch fold using the methylated form of its cognate ligand as cofactor. Additionally, new RNA sequencing methods have been developed to detect this modification in transcriptome-wide manner. For all these reasons, an increasing demand for synthetic m^3^C containing oligoribonucleotides is emerging. Their chemical synthesis relies on RNA solid-phase synthesis using phosphoramidite building blocks. Here, we describe a facile synthetic path towards N^4^-acetylated 2′-*O*-TBDMS- and 2′-*O*-TOM m^3^C phosphoramidites to provide an optimal toolbox for solid-phase synthesis of m^3^C containing RNA.

**Graphical abstract:**

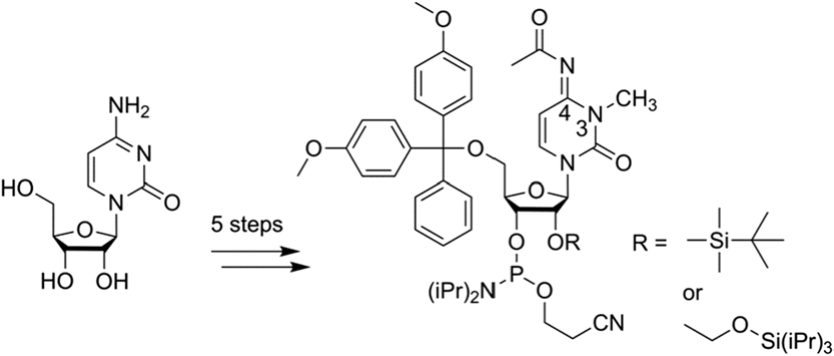

**Supplementary Information:**

The online version contains supplementary material available at 10.1007/s00706-022-02896-x.

## Introduction

More than 170 different nucleoside modifications have been identified in non-coding and coding RNAs [1–4]. These modifications influence formatively the cellular fate of RNAs by modulating their stabilities and functions. The most abundant class of RNA modifications is methylation which occurs in high structural diversity, including ribose 2′-O and almost any position of the four heterocyclic nucleobases, adenine, cytosine, guanine, and uracil. While nucleoside methylations are traditionally associated with tRNA, rRNA, and caps of mRNA, the recent discoveries on reversible mRNA methylation have opened a new realm of post-transcriptional gene regulation. In particular, *N*^6^-methyladenosine (m^6^A) has been disclosed as a modification that eukaryotic cells utilize to tune mRNA metabolism and translation [5, 6]. More recently, 3-methylcytidine (m^3^C) has gained a lot of attention, because evidence for its occurrence in mRNA of mice and humans has been reported [7–9]. Notably, m^3^C was first discovered in 1963 in total RNA of yeast [10] and was later identified in the anticodon loop of eukaryotic tRNA where it impacts fold stability, ribosome-binding affinity and decoding activity of tRNA as well as mRNA processing [11–15]. Very recently, m^3^C has been identified as methylation product of preQ_1_ class I riboswitches possessing ribozyme (methyltransferase) activity in vitro when m^6^preQ_1_ (2-amino-7-aminomethyl-6-methoxy-7-deazapurine) is provided as cofactor [16, 17]. Additionally, new RNA sequencing methods, AlkAniline-Seq [18] and HAC-seq [19], have been developed to detect m^3^C modifications in transcriptome-wide manner. Furthermore, deoxyribozyme tools to detect m^3^C, m^4^C and m^5^C have been selected in vitro and are able to distinguish the methylation position based on distinct kinetic signatures of their RNA catalyzed backbone cleavage reaction at the site of modification [20].

Recently, Mao et al. [21] reported the synthesis of *N*^4^-benzoyl-2′-*O*-*tert*-butyldimethylsilyl(TBDMS)-3-methylcytidine (m^3^C) phosphoramidite along with comprehensive biophysical analysis of short m^3^C containing RNAs. Shortly thereafter, Liaqat et al. [20] synthesized *N*^4^-benzoyl-2′-*O*-[(triisopropylsilyl)oxy]methyl(TOM)-3-methylcytidine for the investigation of RNA cleaving deoxyribozymes. *N*^4^-Benzoyl protected cytidines, however, are troublesome during standard RNA deprotection protocols which generally involve aqueous methylamine. Thereby, the *N*^4^-benzoyl moiety is partially substituted at the C4 atom by methylamine, resulting in N^4^-methylated cytidine derivatives. This problem can usually be circumvented if *N*^4^-acetyl instead of benzoyl protection is applied [22, 23]. Moreover, for 2′-O protection we envisaged both 2′-*O*-TBDMS (to satisfy the most widespread concept of commercially available nucleoside building blocks) [24–27] and 2′-*O*-TOM protection [28–31]. The latter is conducive for the solid-phase synthesis of long RNAs (> 50–60 nt) due to reduced steric hindrance during the coupling step and hence very high coupling yields [28, 29].

## Results and discussion

Our synthesis started from commercially available cytidine which was selectively methylated at position N3 by iodomethane to yield the corresponding hydroiodide salt of m^3^C **1** (Scheme 1) [21]. Subsequently, the 5′ hydroxyl group was masked as dimethoxytrityl ether using dimethoxytrityl chloride to furnish compound **2** [21]. Transient protection of the ribose OH groups as trimethylsilyl ether, followed by N^4^ acetylation using acetyl chloride, subsequent desilylation with methanol and aqueous workup delivered compound **3**. Introduction of the 2′-*O*-TBDMS group was accomplished by treatment with *tert*-butyldimethylsilyl chloride under basic conditions [24–26] resulting in a mixture of 2′ and 3′ regioisomers that was separated by column chromatography, providing compound **4a**. Alternatively, the 2′-*O*-TOM group was attached via in situ formation of a 2′,3′-*O*-di-*tert*-butylstannylidene complex [30]. This cyclic intermediate was then treated with (triisopropylsiloxy)methyl chloride yielding a mixture of 2′ and 3′ regioisomers that was separated by column chromatography, providing compound **4b**. Finally, phosphitylation was executed with 2-cyanoethyl-*N*,*N*-diisopropylchlorophosphoramidite under basic conditions. Starting with cytidine, our route provided nucleosides **5a** and **5b** in 21% and 25% overall yields in five steps and with four chromatographic purifications; in total, 0.5 g of **5a** and 0.4 g of **5b** were obtained in the course of this study.

**Figure:**
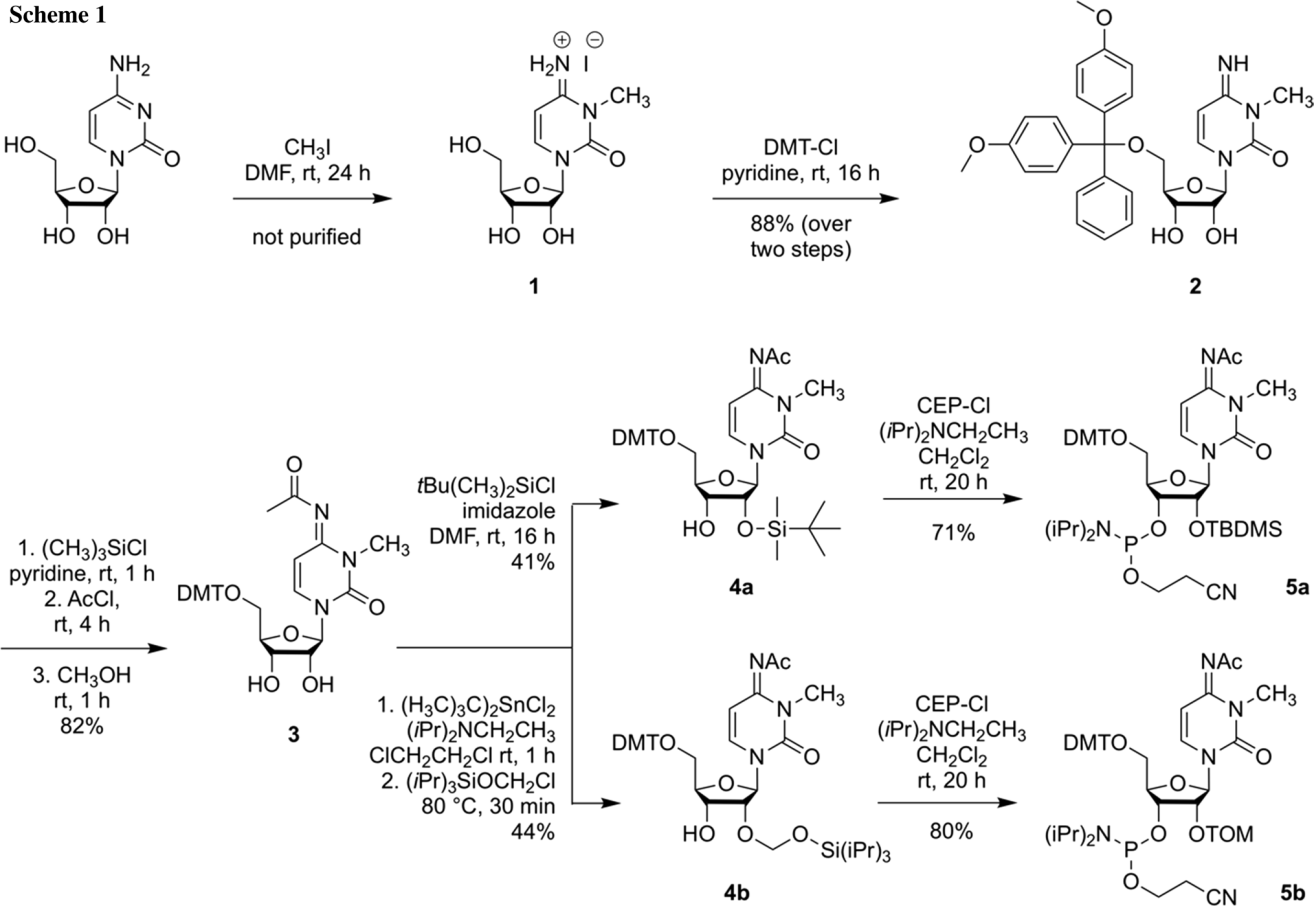

**Fig. 1:**
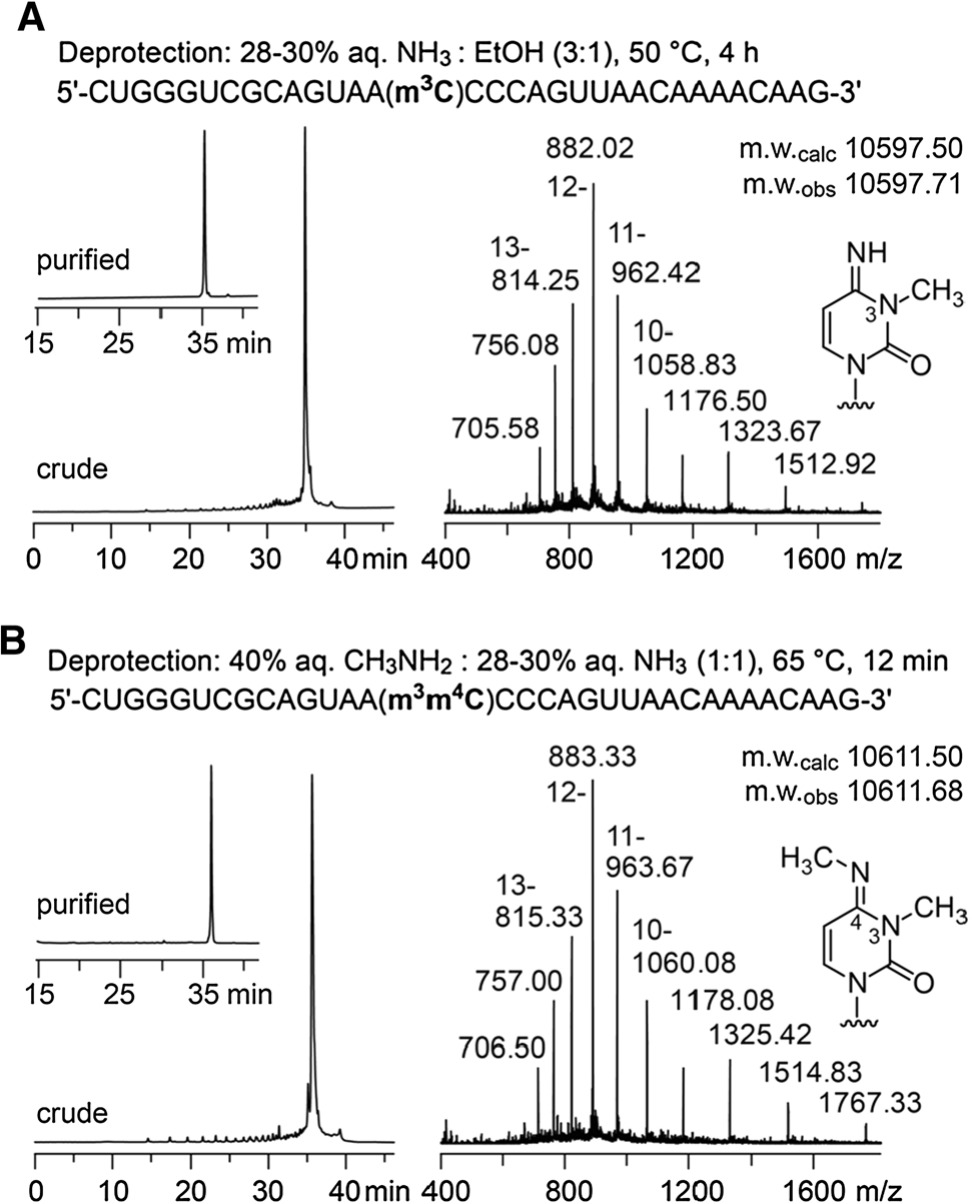
HPLC and mass spectrometric analysis of synthetic RNA using the novel m^3^C building blocks **5a** and **5b**. **A** Deprotection of a 33 nt RNA using ammonia. Anion exchange chromatogram of crude deprotected RNA (inset shows the RNA after purification); LC–ESI–MS confirmed m^3^C modified RNA as major product. HPLC conditions: Dionex DNAPac PA100, 4 × 250 mm, at 80 °C; solvent A was 25 mM Tris–HCl (pH 8.0) and 20 mM NaClO_4_ in 20% aqueous acetonitrile; solvent B was 25 mM Tris–HCl (pH 8.0) and 0.6 M NaClO_4_ in 20% aqueous acetonitrile; the gradient was: linear, 0–60% with slope of 5% solvent B per column volume). **B** Same as **A**, but deprotection using aqueous methylamine and ammonia (‘AMA’). Transamination provides m^3^m^4^C modified RNA as major product consistent with LC–ESI–MS analysis

The solid-phase synthesis of RNA with site-specific m^3^C modifications (using the novel building blocks **5a** and **5b**) was performed following standard RNA synthesis protocols (see Supporting Information and references [24–29]). Coupling yields of the novel building blocks were higher than 98% according to the trityl assay. Cleavage of the oligonucleotides from the solid support and their deprotection were performed using aqueous ammonium hydroxide in ethanol followed by treatment with tetra-*n*-butylammonium fluoride in tetrahydrofuran. Salts were removed by size-exclusion chromatography on a Sephadex G25 column, and RNA sequences were purified by anion-exchange chromatography under denaturating conditions (for a typical example see Fig. 1A, left panel). The molecular weights of the purified oligoribonucleotides were confirmed by liquid-chromatography (LC) electrospray-ionization (ESI) mass spectrometry (MS) (Fig. 1A, right panel). Importantly, when aqueous CH_3_NH_2_ and NH_3_ (‘AMA’ deprotection) was used for RNA deprotection, we obtained double methylated RNA as major product (for a typical example see Fig. 1B, left panel) that was assigned to the transaminated m^3^m^4^C modified RNA by LC–ESI–MS (Fig. 1B, right panel).

## Conclusion

The growing evidence for 3-methylated cytidines playing important roles in the life cycle of cellular RNA entails an increasing demand for synthetic m^3^C modified oligoribonucleotides. These are needed for diverse applications ranging from simple RNA referencing to method developments aiming at advanced m^3^C RNA sequencing approaches. The here presented synthesis of 2′-*O*-TBDMS- and 2′-*O*-TOM protected m^3^C phosphoramidites **5a** and **5b** with *N*^*4*^-acetyl protection of the nucleobase is practical and high-yielding. The novel building blocks are directly applicable in standard coupling cycles for RNA solid-phase synthesis. Importantly, it has to be taken into account that for RNA deprotection, transamination at the N^4^-acylated m^3^C nucleobase was observed if methylamine was applied. Therefore, RNA deprotection using ammonia is a requirement to accomplish the chemical synthesis of m^3^C modified RNA in high quality.

More generally speaking, we point out that finetuning of protection groups in RNA solid-phase synthesis is needed to push the limits of accessible RNA lengths (> 50–60 nt). Thereby, the critical step is not the assembly of the RNA on the solid support but deprotection of the RNA, and hence, more labile acetyl protection of nucleobase exocyclic amino groups is preferred over benzoyl protection. In particular, an additional advantage for the application of *N*^4^-acetylcytidine instead of *N*^4^-benzoylcytidine building blocks is that transamination at C4 is avoided under standard RNA deprotection conditions which involve aqueous methylamine solutions. However, in the case of 3-methylcytidine building blocks, the expectation that *N*^4^-acetyl protection also eliminates transamination did not fulfill in our hands; deprotection procedures based on ammonia instead of methylamine are required. Our findings, therefore, help to resolve contrasting reports in the literature upon suitable basic deprotection conditions for synthetic m^3^C modified RNA [20, 21].

## Experimental

Unless stated otherwise, all reactions were carried out under argon atmosphere using absolute solvents. Solvents and other reagents were purchased in highest quality from commercial suppliers (Sigma-Aldrich, Carbosynth, ChemGenes) and were used without further purification. ^1^H and ^13^C spectra were recorded on a Bruker DRX 400 MHz spectrometer. Chemical shifts (*δ*) are reported relative to tetramethylsilane (TMS) and were referenced to the residual signal of the deuterated solvent (CDCl_3_: 7.26 ppm for ^1^H and 77.16 ppm for ^13^C; DMSO-*d*_*6*_: 2.50 ppm for ^1^H and 39.52 ppm for ^13^C). Signals were assigned according to ^1^H-^1^H-COSY, ^1^H-^13^C-HSQC, and ^1^H-^13^C-HMBC experiments. Following abbreviations are used to describe observed multiplicity: s = singlet, d = doublet, t = triplet, m = multiplet and br = broad. Diastereomeric protons which appear as distinct signals are marked with index a and b. Identity of synthesized compounds was further confirmed by high-resolution mass spectrometry experiments using a Thermo Scientific Q Exacative Orbitrap with an electrospray ion source. MS data were collected in the positive ion mode. Reaction progress was monitored via thin layer chromatography (TLC, Macherey–Nagel) with fluorescent indicator. Column chromatography was carried out on silica gel 60 (70–230 mesh).

### 3-Methylcytidinium iodide (1, C_10_H_16_N_3_O_5_)

In analogy to Ref. [21]. Cytidine (1.70 g, 7.00 mmol) was suspended in 17.5 cm^3^ *N*,*N*-dimethylformamide and treated with 0.87 cm^3^ iodomethane (2.0 eq, 13.97 mmol). After 24 h, the solvent was removed under high vacuum and the residue was coevaporated three times with toluene. The solid was used in the next step without further purification. TLC (25% MeOH in CH_2_Cl_2_): *R*_*f*_ = 0.60; HR-ESI–MS: *m/z* calculated for [C_10_H_16_N_3_O_5_]^+^ ([M]^+^) 258.1084, found 258.1079; ^1^H NMR (DMSO-*d*_*6*_, 400 MHz): *δ* = 3.35 (3H, s, CH_3_ (N3)), 3.60 (1H, dd, ^2^*J*_HH_ = 12.35 Hz, ^3^*J*_HH_ = 1.65 Hz, CH_a_ (5′)), 3.73 (1H, dd, ^2^*J*_HH_ = 12.74 Hz, ^3^*J*_HH_ = 1.71 Hz, CH_b_ (5′)), 3.90–3.96 (2H, m, CH (3′), CH (4′)), 4.03 (1H, t, ^3^*J*_HH_ = 3.39 Hz, CH (2′)), 5.16, 5.51 (3H, 2 × s, OH (2′), OH (3′), OH (5′)), 5.71 (1H, d, ^3^*J*_HH_ = 2.98 Hz, CH (1′)), 6.19 (1H, d, ^3^*J*_HH_ = 7.89 Hz, CH (5)), 8.32 (1H, d, ^3^*J*_HH_ = 7.89 Hz, CH (6)), 9.15, 9.78 (2H, 2 × s, 2 × NH) ppm; ^13^C NMR (DMSO-*d*_*6*_, 101 MHz): *δ* = 30.73 CH_3_ (N3), 59.71 CH_2_ (5′), 68.56 C (3′), 74.14 C (2′), 84.62 C (4′), 90.71 C (1′), 94.07 C (5), 141.62 C (6), 147.63 C (2), 158.93 C (4) ppm.

### 5′-*O*-(4,4′-Dimethoxytrityl)-3-methylcytidine (2, C_31_H_33_N_3_O_7_)

In analogy to Ref. [21]. Compound **1** (7.00 mmol) was coevaporated with pyridine and was subsequently dried under high vacuum for 30 min at 60 °C. It was then dissolved in 20.6 cm^3^ pyridine, and 3.558 g 4,4′-dimethoxytrityl chloride (1.5 eq, 10.5 mmol) was added in two portions over a period of 30 min. The reaction mixture was stirred at ambient temperature for 16 h, was then quenched by the addition of methanol and solvents were evaporated. The oily residue was coevaporated with toluene and was then dissolved in dichloromethane. Extraction with 5% sodium thiosulfate solution was followed by extraction with saturated sodium bicarbonate solution and brine. The organic layer was dried over sodium sulfate, the solvent was evaporated, and the crude product was purified by flash chromatography on deactivated silica gel (4–10% MeOH in CH_2_Cl_2_). Yield: 3.678 g of **2** as a white foam (88% over 2 steps); TLC (10% MeOH in CH_2_Cl_2_): *R*_*f*_ = 0.14; HR-ESI–MS: *m/z* calculated for [C_31_H_34_N_3_O_7_]^+^ ([M + H]^+^) 560.2391, found 560.2381; ^1^H NMR (CDCl_3_, 400 MHz): *δ* = 3.34 (3H, s, CH_3_ (N3)), 3.41 (1H, dd, ^2^*J*_HH_ = 10.78 Hz, ^3^*J*_HH_ = 2.90 Hz, CH_a_ (5′)), 3.51 (1H, dd, ^2^*J*_HH_ = 10.86 Hz, ^3^*J*_HH_ = 2.47 Hz, CH_b_ (5′)), 3.78 (6H, s, 2 × OCH_3_ (DMT)), 4.18 (1H, m, CH (4′)), 4.27 (1H, t, ^3^*J*_HH_ = 4.18 Hz, CH (2´), 4.37 (1H, t, ^3^*J*_HH_ = 5.11 Hz, CH (3′)), 5.37 (1H, d, ^3^*J*_HH_ = 8.08 Hz, CH (5)), 5.89 (1H, d, ^3^*J*_HH_ = 3.80 Hz, CH (1′)), 6.84, 7.21–7.31 (11H, m, aromat. CH (DMT)), 7.39–7.41 (3H, m, aromat. CH (DMT), CH (6)) ppm; ^13^C NMR (CDCl_3_, 101 MHz): *δ* = 29.23 CH_3_ (N3), 55.34 2 × OCH_3_ (DMT), 62.65 C (5′), 70.55 C (3′), 75.31 C (2′), 83.94 C (4′), 86.97 aromat. C (DMT), 90.67 C (1′), 101.15 C (5), 113.33, 127.15, 128.04, 128.24, 130.21, 130.25 aromat C (DMT), 132.41 C (6), 135.39, 135.50, 144.52 aromat. C (DMT), 151.04 C (2), 158.65 C (4), 158.72 aromat. C (DMT) ppm.

### *N*^4^-Acetyl-5′-*O*-(4,4′-dimethoxytrityl)-3-methylcytidine (3, C_33_H_35_N_3_O_8_)

Compound **2** (1.275 g, 2.28 mmol) was coevaporated trice with pyridine and was subsequently dried under high vacuum overnight. It was then dissolved in 28.3 cm^3^ pyridine and was treated with 1.16 cm^3^ chlorotrimethylsilane (4.0 eq, 9.11 mmol) over a period of 5 min. The reaction mixture was allowed to stir for 1 h followed by the dropwise addition of 0.19 cm^3^ acetyl chloride (1.2 eq, 2.73 mmol) over 10 min. Stirring was continued for 4 h, followed by addition of 20 cm^3^ methanol. Solvents were removed after an additional hour of stirring, and the residue was taken up in ethyl acetate. Extraction with saturated sodium bicarbonate solution and brine was followed by drying of the organic phase over sodium sulfate. After evaporation, the crude product was purified by column chromatography on silica gel (0–4% MeOH in CH_2_Cl_2_). Yield: 1.125 mg of **3** as a white foam (82%); TLC (5% MeOH in CH_2_Cl_2_): *R*_*f*_ = 0.47; HR-ESI–MS: *m/z* calculated for [C_33_H_36_N_3_O_8_]^+^ ([M + H]^+^) 602.2497, found 602.2489; ^1^H NMR (CDCl_3_, 400 MHz): *δ* = 2.20 (3H, s, OAc (N^4^)), 3.22 (1H, d, ^3^*J*_HH_ = 4.94 Hz, OH (3´)), 3.37 (3H, s, CH_3_ (N3)), 3.40 (1H, dd, ^2^*J*_HH_ = 10.96 Hz, ^3^*J*_HH_ = 3.19 Hz, CH_a_ (5′)), 3.49 (1H, dd, ^2^*J*_HH_ = 10.98 Hz, ^3^*J*_HH_ = 2.74 Hz, CH_b_ (5′)), 3.80 (6H, s, 2 × OCH_3_ (DMT)), 4.22 (1H, m, (4′)), 4.26 (1H, m, CH (2′)), 4.31 (1H, m, OH (2′)), 4.36 (1H, dd, ^3^*J*_HH_ = 9.69 Hz, ^3^*J*_HH_ = 4.81 Hz, CH (3′)), 5.81 (1H, d, ^3^*J*_HH_ = 3.45 Hz, CH (1′)), 5.96 (1H, d, ^3^*J*_HH_ = 8.21 Hz, CH (5)), 6.83–6.85, 7.24–7.38 (13H, m, aromat. CH (DMT)), 7.62 (1H, d, ^3^*J*_HH_ = 8.21 Hz, CH (6)) ppm; ^13^C NMR (CDCl_3_, 101 MHz): *δ* = 27.29 OAc (N^4^), 29.74 CH_3_ (N3), 55.37 2 × OCH_3_ (DMT), 62.44 C (5′), 70.81 C (3′), 76.31 C (2′), 84.51 C (4′), 87.17 aromat. C (DMT), 91.61 C (1′), 97.65 C (5), 113.40, 127.25, 128.11, 128.22, 130.15, 130.20 aromat. C (DMT), 135.29 C (6), 135.39, 135.47, 144.26 aromat. C (DMT), 151.29 C (2), 153.26 C (4), 158.79, 158.81 aromat. C (DMT), 184.41 C = O (OAc N^4^) ppm.

### *N*^4^-Acetyl-2′-*O*-*tert*-butyldimethylsilyl-5′-*O*-(4,4′-dimethoxytrityl)-3-methylcytidine (4a, C_39_H_49_N_3_O_8_Si)

Imidazole (2.0 eq, 1.80 mmol, 123 mg) and 163 mg *tert*-butyldimethylsilyl chloride (1.2 eq, 1.08 mmol) were added consecutively to a solution of 542 mg compound **3** (0.90 mmol) in 5.4 cm^3^ *N*,*N*-dimethylformamide and stirred for 16 h. Then, solvents were removed, the residue was taken up in ethyl acetate and was washed extensively with brine. The organic layer was dried over sodium sulfate, was concentrated and the solid purified by column chromatography on silica gel (10–30% ethyl acetate in cyclohexane). Yield: 267 mg of **4a** as a white foam (41%); TLC (3% MeOH in CH_2_Cl_2_): *R*_*f*_ = 0.50; HR-ESI–MS: *m/z* calculated for [C_39_H_50_N_3_O_8_Si]^+^ ([M + H]^+^) 716.3362, found 716.3353; ^1^H NMR (DMSO-*d*_*6*_, 400 MHz): *δ* = 0.076, 0.092 (6H, 2, s, 2 × CH_3_ (TBDMS)), 0.87 (9H, s, *t*-Bu (TBDMS)), 2.09 (3H, s, OAc (N^4^)), 3.23 (3H, s, CH_3_ (N3)), 3.29 (1H, dd, ^2^*J*_HH_ = 11.02 Hz, ^3^*J*_HH_ = 2.25 Hz, CH_a_ (5′)), 3.32–3.36 (1H, m, CH_b_ (5′)), 3.75 (6H, s, 2 × OCH_3_ (DMT)), 4.01 (1H, m, (4′)), 4.14 (1H, m, CH (3′)), 4.20 (1H, m, CH (2′)), 5.16 (1H, d, ^3^*J*_HH_ = 6.29 Hz, OH (3′)), 5.70 (1H, d, ^3^*J*_HH_ = 2.37 Hz, CH (1′)), 5.82 (1H, d, ^3^*J*_HH_ = 8.17 Hz, CH (5)), 6.89–6.91, 7.23–7.29 (13H, m, aromat. CH (DMT)), 7.25 (1H, d, ^3^*J*_HH_ = 8.19 Hz, CH (6)) ppm; ^13^C NMR (DMSO-*d*_*6*_, 101 MHz): *δ* = − 5.00, − 4.66 2 × CH_3_ (TBDMS), 17.96 C_q_ (TBDMS), 25.70 *t*-Bu (TBDMS), 27.13 OAc (N^4^), 29.26 CH_3_ (N3), 55.04 2 × OCH_3_ (DMT), 61.89 C (5′), 68.57 C (3′), 75.89 C (2′), 81.92 C (4′), 86.00 aromat. C (DMT), 90.31 C (1′), 96.26 C (5), 113.37, 126.87, 127.79, 127.94, 129.73, 129.80, 135.10, 135.38 aromat. C (DMT), 135.94 C (6), 144.39 aromat. C (DMT), 149.65 C (2), 152.93 C (4), 158.18 aromat. C (DMT), 182.66 C = O (OAc N^4^) ppm.

### *N*^4^-Acetyl-5′-*O*-(4,4′-dimethoxytrityl)-2′-*O*-[[(triisopropylsilyl)oxy]methyl]-3-methylcytidine (4b, C_43_H_57_N_3_O_9_Si)

*N*,*N*-Diisopropylethylamine (3.5 eq, 2.94 mmol, 0.51 cm^3^) and 281 mg di-*tert*-butyltin dichloride (1.1 eq, 0.92 cm^3^) were added to a solution of 505 mg compound **3** (0.84 mmol) in 6.5 cm^3^ dichloroethane. The reaction mixture was stirred 1 h at ambient temperature and was then heated to 80 °C. (Triisopropylsiloxy)methyl chloride (1.3 eq, 1.03 mmol, 0.25 cm^3^) was added dropwise and stirring was continued at 80 °C for 30 min. The dark solution was cooled to ambient temperature, was diluted with dichloromethane and was washed with saturated sodium bicarbonate solution. The crude product was purified by column chromatography on silica gel (10–50% ethyl acetate in cyclohexane). Yield: 291 mg of **4b** as a white foam (44%); TLC (1% MeOH in CH_2_Cl_2_): *R*_*f*_ = 0.42; HR-ESI–MS: *m/z* calculated for [C_43_H_58_N_3_O_9_Si]^+^ ([M + H]^+^) 788.3937, found 788.3918; ^1^H NMR (DMSO-*d*_*6*_, 400 MHz): *δ* = 0.96–1.05 (21H, m, Si(CH(CH_3_)_2_)_3_), 2.09 (3H, s, Ac (N^4^)), 3.21 (3H, s, CH_3_ (N3)), 3.27 (2H, m, CH_2_ (5′)), 3.74 (6H, s, 2 × OCH_3_ (DMT)), 4.00 (1H, m, CH (4′)), 4.15 (1H, quartettoid, ^3^*J*_HH_ = 5.32 Hz, CH (3′)), 4.27 (1H, t, ^3^*J*_HH_ = 5.08 Hz, CH (2′)), 4.93 (1H, d, ^3^*J*_HH_ = 5.20 Hz, CH_a_ (TOM)), 5.00 (1H, d, ^3^*J*_HH_ = 5.17 Hz, CH_b_ (TOM)), 5.29 (1H, d, ^3^*J*_HH_ = 6.00 Hz, OH (3′)), 5.91 (1H, d, ^3^*J*_HH_ = 8.17 Hz, CH (5)), 5.94 (1H, d, ^3^*J*_HH_ = 4.88 Hz, CH (1′)), 6.88–6.90, 7.22–7.38 (13H, m, aromat. CH (DMT)), 7.59 (1H, d, ^3^*J*_HH_ = 8.19 Hz, CH (6)) ppm; ^13^C NMR (DMSO-*d*_*6*_, 101 MHz): *δ* = 11.35 Si(*C*H(CH_3_)_2_)_3_), 17.58 Si(CH(*C*H_3_)_2_)_3_), 27.01 Ac (N^4^), 29.27 CH_3_ (N3), 55.01 2 × OCH_3_ (DMT), 62.90 C (5′), 68.43 C (3′), 77.77 C (2′), 83.39 C (4′), 86.05 aromat. C (DMT), 87.66 C (1′), 88.48 CH_2_ (TOM), 96.80 C (5), 113.23, 126.81, 127.75, 127.90, 129.72, 129.76, 135.20, 135.38 aromat. C (DMT), 136.21 C (6), 144.43 aromat. C (DMT), 149.72 C (2), 152.24 C (4), 158.14 aromat. C (DMT), 182.67 C = O (Ac N^4^) ppm.

### *N*^4^-Acetyl-2′-*O*-*tert*-butyldimethylsilyl-5′-*O*-(4,4′-dimethoxytrityl)-3-methylcytidine 3′-*O*-(2-cyanoethyl-*N*,*N*-diisopropylphosphoramidite) (5a, C_48_H_66_N_5_O_9_PSi)

Compound **4a** (267 mg, 0.37 mmol) was dried under high vacuum overnight. It was then dissolved in 4.0 cm^3^ dichloromethane and consecutively treated with 0.26 cm^3^ *N*,*N*-diisopropylethylamine (4.0 eq, 1.49 mmol) and 177 mg 2-cyanethyl-*N*,*N*-diisopropylchlorophosphoramidite (2.0 eq, 0.75 mmol). After 20 h, the reaction mixture was diluted with dichloromethane and was washed with 5% sodium bicarbonate solution. The combined organic layers were dried over sodium sulfate and the crude product was purified by column chromatography on silica gel (10–30% ethyl acetate in cyclohexane + 1% Et_3_N). Yield: 243 mg of **5a** as a white foam (71%); TLC (3% MeOH in CH_2_Cl_2_): *R*_*f*_ = 0.40 (both diastereomers); HR-ESI–MS: *m/z* calculated for [C_48_H_67_N_5_O_9_PSi]^+^ ([M + H]^+^) 916.4440, found 916.4429; ^1^H NMR (CDCl_3_, 400 MHz): *δ* = 0.12, 0.14, 0.153, 0.157 (6H, 2 × s, 2 × CH_3_ (TBDMS)), 0.89, 0.90 (9H, s, *t*-Bu (TBDMS)), 0.99 (3H, d, ^3^*J*_HH_ = 7.00 Hz, N(CH(C***H***_3_)_2_)_2_), 1.15 (9H, d, ^3^*J*_HH_ = 6.64 Hz, N(CH(C***H***_3_)_2_)_2_), 2.18, 2.19 (3H, s, Ac (N^4^)), 2.38 (1H, 2 × t, ^3^*J*_HH_ = 6.46, 6.44 Hz, CH_a_CN), 2.63 (1H, 2 × t, ^3^*J*_HH_ = 6.16, 6.21 Hz, CH_b_CN), 3.35, 3.36 (3H, s, CH_3_ (N3)), 3.31–3.41 (1H, m, CH_a_ (5′)), 3.50–3.71 (4H, m, CH_b_ (5′), N(C***H***(CH_3_)_2_)_2_), POCH_a_), 3.806, 3.812 (6H, s, 2 × OCH_3_ (DMT)), 3.76–3.96 (1H, m, POCH_b_), 4.22–4.35 (3H, m, CH (2′), CH (3′), (4′)), 5.75 (0.5H, d, ^3^*J*_HH_ = 8.16 Hz, CH_a_ (5)), 5.81 (0.5H, d, ^3^*J*_HH_ = 8.20 Hz, CH_b_ (5)), 5.88 (0.5H, d, ^3^*J*_HH_ = 2.52 Hz, CH_a_ (1′)), 5.96 (0.5H, d, ^3^*J*_HH_ = 4.01 Hz, CH_b_ (1′)), 6.82–6.86, 7.25–7.41 (13H, m, aromat. CH (DMT)), 7.72 (0.5H, d, ^3^*J*_HH_ = 8.19 Hz, CH_a_ (6)), 7.81 (0.5H, d, ^3^*J*_HH_ = 8.19 Hz, CH_b_ (6)) ppm; ^13^C NMR (CDCl_3_, 101 MHz): *δ* = − 4.73, − 4.71, − 4.61, − 4.59, − 4.51, − 4.46 2 × CH_3_ (TBDMS), 18.13, 18.17 C_q_ (TBDMS), 20.31 (d, ^3^*J*_CP_ = 7.11 Hz, CH_2_CN_a_), 20.59 (d, ^3^*J*_CP_ = 6.54 Hz, CH_2_CN_b_), 24.65, 24.70, 24.72, 24.78, 24.86, 24.94 N(CH(***C***H_3_)_2_)_2_), 25.84, 25.88 *t*-Bu (TBDMS), 27.04, 27.29 OAc (N^4^), 29.73, 29.76 CH_3_ (N3), 43.12 (d, ^2^*J*_CP_ = 12.35 Hz, N(***C***_a_H(CH_3_)_2_)_2_), 43.43 (d, ^2^*J*_CP_ = 13.08 Hz, N(***C***_b_H(CH_3_)_2_)_2_), 55.36, 55.39 2 × OCH_3_ (DMT), 57.94 (d, ^2^*J*_CP_ = 21.07 Hz, POC_a_H_2_), 58.51 (d, ^2^*J*_CP_ = 17.44 Hz, POC_b_H_2_), 61.86, 62.31 C (5′), 71.44 (d, ^2^*J*_CP_ = 9.45 Hz, C_a_ (3′)), 72.06 (d, ^2^*J*_CP_ = 15.99 Hz, C_b_ (3′)), 75.32 (d, ^3^*J*_CP_ = 3.63 Hz, C_a_ (2′)), 75.92 (d, ^3^*J*_CP_ = 2.18 Hz, C_b_ (2′)), 82.37 C (4′), 87.20, 87.34 aromat. C (DMT), 89.57, 90.02 C (1′), 97.41, 97.47 C (5), 113.35, 113.41 aromat. C (DMT), 117.45, 117.70 CN, 127.31, 128.09, 128.14, 128.39, 128.46, 130.32, 130.36, 130.40, 135.34, 135.70 aromat. C (DMT), 135.12, 135.50 C (6), 144.18, 144.32 aromat. C (DMT), 150.45, 150.50 C (2), 153.26, 153.32 C (4), 158.85 aromat. C (DMT), 184.21 C = O (Ac N^4^) ppm; ^31^P NMR (CDCl_3_, 162 MHz): *δ* = 149.70, 149.97 ppm.

### *N*^4^-Acetyl-5′-*O*-(4,4′-dimethoxytrityl)-2′-*O*-[[(triisopropylsilyl)oxy]methyl]-3-methylcytidine 3′-*O*-(2-cyanoethyl-*N*,*N*-diisopropylphosphoramidite) (5b, C_52_H_74_N_5_O_10_PSi)

Compound **4b** (328 mg, 0.42 mmol) was dissolved in 2.8 cm^3^ dichloromethane and was consecutively treated with 0.18 cm^3^ *N*,*N*-diisopropylethylamine (2.5 eq, 1.04 mmol) and 148 mg 2-cyanethyl-*N*,*N*-diisopropylchlorophosphoramidite (1.5 eq, 0.62 mmol). The colorless solution was stirred for 20 h at ambient temperature, was then diluted with dichloromethane and washed with 5% sodium bicarbonate solution. The crude product was purified by column chromatography on silica gel (10–40% ethyl acetate in cyclohexane + 1% Et_3_N). Yield: 328 mg of **5b** as a white foam (80%); TLC (1% MeOH in CH_2_Cl_2_): *R*_*f*_ = 0.33 (both diastereomers); HR-ESI–MS: *m/z* calculated for [C_52_H_75_N_5_O_10_PSi]^+^ ([M + H]^+^) 988.5015, found 988.5001; ^1^H NMR (CDCl_3_, 400 MHz): *δ* = 1.00–1.04, 1.14–1.17 (33H, m, Si(CH(CH_3_)_2_)_3_, N(CH(C***H***_3_)_2_)_2_), 2.18, 2.19 (3H, s, Ac (N^4^)), 2.37 (1H, 2 × t, ^3^*J*_HH_ = 6.39, 6.44 Hz, CH_a_CN), 2.63 (1H, m, CH_b_CN), 3.34, 3.35 (3H, s, CH_3_ (N3)), 3.37 (1H, m, CH_a_ (5′)), 3.50–3.67 (4H, m, CH_b_ (5′), N(C***H***(CH_3_)_2_)_2_, POCH_a_), 3.80, 3.81 (6H, s, 2 × OCH_3_ (DMT)), 3.80–3.99 (1H, m, POCH_b_), 4.19 (0.5H, m, CH_a_ (4′)), 4.25 (0.5H, m, CH_b_ (4′)), 4.33–4.47 (2H, m, CH (2′), CH (3′)), 5.05 (2H, 4 × d, CH_2_ (TOM)), 5.81 (0.5H, d, ^3^*J*_HH_ = 8.19 Hz, CH_a_ (5)), 5.86 (0.5H, d, ^3^*J*_HH_ = 8.19 Hz, CH_b_ (5)), 6.13 (0.5H, d, ^3^*J*_HH_ = 4.29 Hz, CH_a_ (1′)), 6.15 (0.5H, d, ^3^*J*_HH_ = 4.37 Hz, CH_b_ (1)), 6.82–6.85, 7.25–7.41 (13H, m, aromat. CH (DMT)), 7.59 (0.5H, d, ^3^*J*_HH_ = 8.21 Hz, CH_a_ (6)), 7.66 (0.5H, d, ^3^*J*_HH_ = 8.21 Hz, CH_b_ (6)) ppm; ^13^C NMR (CDCl_3_, 101 MHz): *δ* = 12.05, 12.07 Si(*C*H(CH_3_)_2_)_3_), 17.91, 17.94 Si(CH(*C*H_3_)_2_)_3_), 20.28 (d, ^3^*J*_CP_ = 7.27 Hz, CH_2_CN_a_), 20.51 (d, ^3^*J*_CP_ = 6.54 Hz, CH_2_CN_b_), 24.61, 24.68, 24.75 N(CH(*C*H_3_)_2_)_2_), 27.04, 27.28 Ac (N^4^), 29.71, 29.74 CH_3_ (N3), 43.29 (d, ^2^*J*_CP_ = 13.08 Hz, N(*C*_a_H(CH_3_)_2_)_2_), 43.46 (d, ^2^*J*_CP_ = 12.35 Hz, N(*C*_b_H(CH_3_)_2_)_2_), 55.35, 55.37 2 × OCH_3_ (DMT), 57.96 (d, ^2^*J*_CP_ = 19.61 Hz, POC_a_H_2_), 58.96 (d, ^2^*J*_CP_ = 16.61 Hz, POC_b_H_2_), 61.99, 62.39 C (5′), 70.32 (d, ^2^*J*_CP_ = 16.71 Hz, C_a_ (3′)), 70.79 (d, ^2^*J*_CP_ = 13.81 Hz, C_b_ (3′)), 77.77 (d, ^3^*J*_CP_ = 4.36 Hz, C_a_ (2′)), 78.40 (d, ^2^*J*_CP_ = 3.46 Hz, C_b_ (2′)), 83.14 (d, ^3^*J*_CP_ = 2.91 Hz, C_a_ (4′)), 83.29 (d, ^2^*J*_CP_ = 2.91 Hz, C_b_ (4′)), 87.13, 87.17 aromat. C (DMT), 87.20, 88.12 C (1′), 89.18, 89.40 CH_2_ (TOM), 97.64, 97.69 C (5), 113.37 aromat. CH (DMT), 117.47, 117.79 CN, 127.27, 128.10, 128.36, 128.42, 128.45, 130.23–130.36 aromat. C (DMT), 135.26–135.74 (m, aromat. C (DMT), C (6)), 144.18, 144.34 aromat. C (DMT), 150.36, 150.40 C (2), 153.11, 153.20 C (4), 158.82 aromat. C (DMT), 184.23 C = O (Ac N^4^) ppm; ^31^P NMR (CDCl_3_, 162 MHz): *δ* = 150.11, 150.64 ppm (2 diastereomers).

## Supplementary Information

Below is the link to the electronic supplementary material.

Supplementary file1 (PDF 2540 KB)
