# Supplementary material for: Synthesis of N4-acetylated 3-methylcytidine phosphoramidites for RNA solid-phase synthesis
Source: Monatsh Chem. 2022 Feb 22;153(3):285–91. doi: 10.1007/s00706-022-02896-x (PMC8948120; doi:10.1007/s00706-022-02896-x)
Supplement: Supplementary file 1 — Supplementary file1 (PDF 2540 KB) [file 706_2022_2896_MOESM1_ESM.pdf]

Supporting Information

to

Synthesis of N^4-acetylated 3-methylcytidine phosphoramidites for RNA solid-phase synthesis

Sarah Moreno[1] ● Laurin Flemmich[1] ● Ronald Micura[1]

[1]Institute of Organic Chemistry and Center for Molecular Biosciences, University of Innsbruck, Austria

Contents

1. NMR spectra of compound **1** 2
2. NMR spectra of compound **2** 3
3. NMR spectra of compound **3** 4
4. NMR spectra of compound **4a** 5
5. NMR spectra of compound **4b** 6
6. NMR spectra of compound **5a** 7
7. NMR spectra of compound **5b** 9
8. RNA solid-phase synthesis of m^3C modified RNA 11
9. Deprotection and purification of m^3C modified RNA 11
10. Mass spectrometry of oligoribonucleotides 12

NMR spectra of compound 1

^{1}H-NMR (400 MHz, d_6-DMSO)

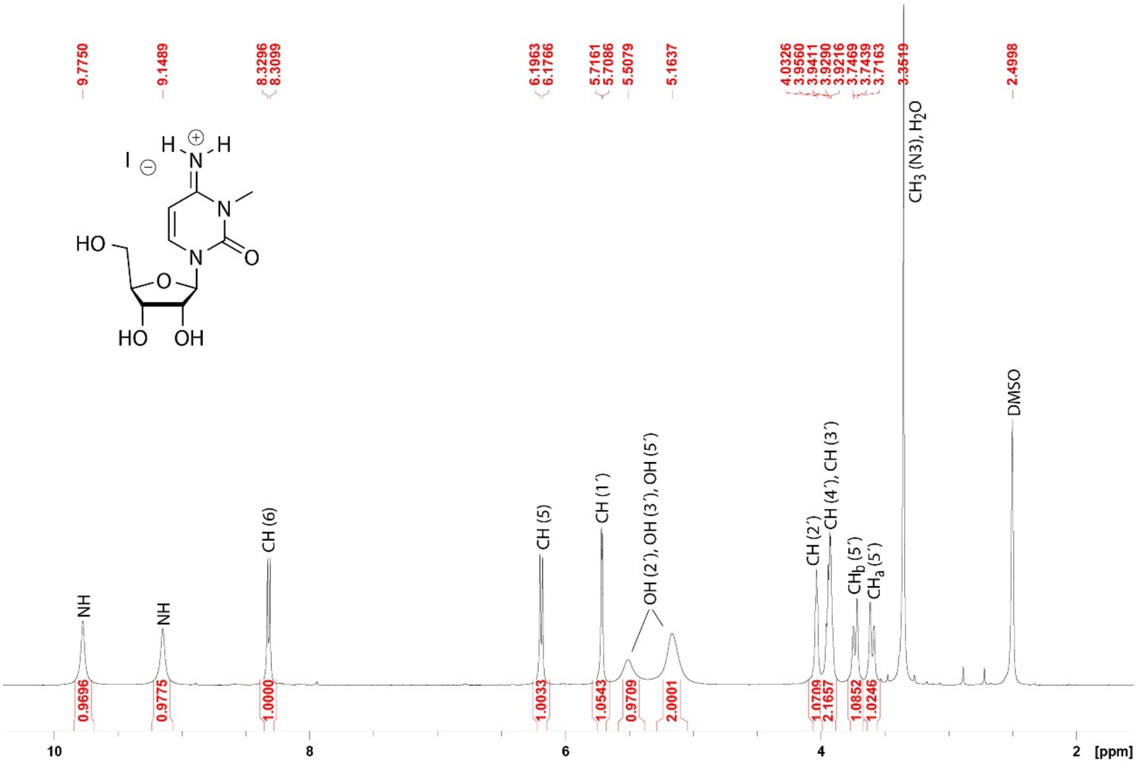

^{13}C-NMR (101 MHz, d_6-DMSO)

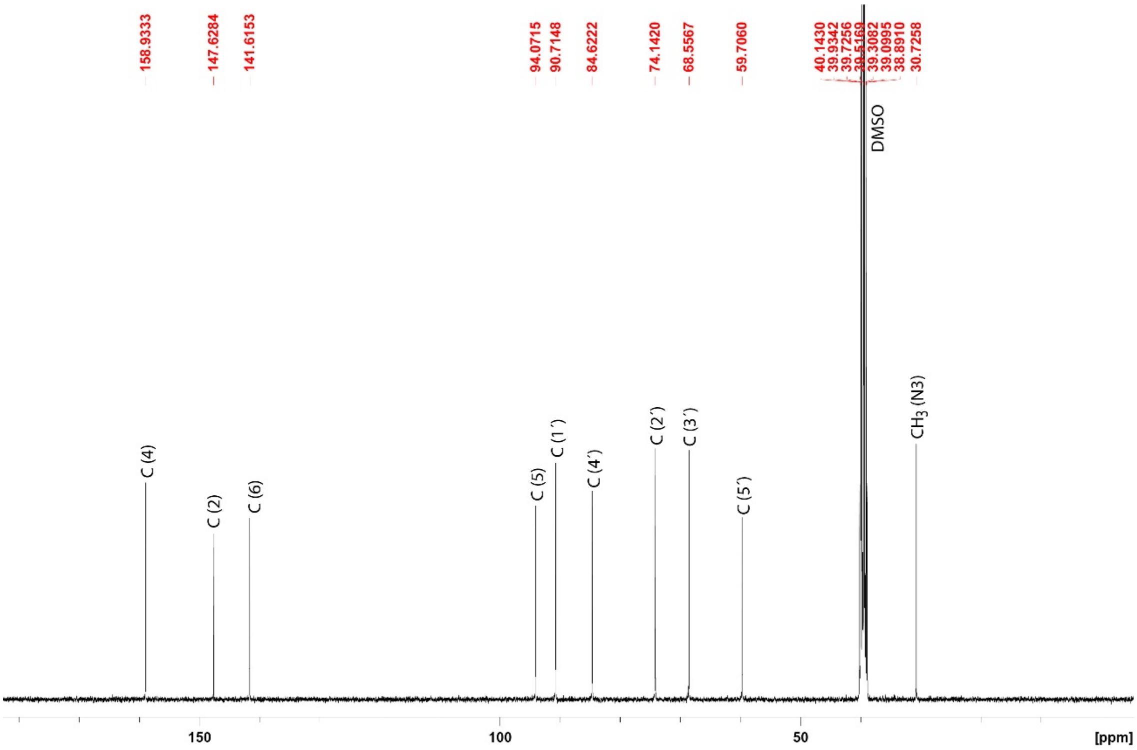

NMR spectra of compound 2

^{1}H-NMR (400 MHz, $CDCl_3$)

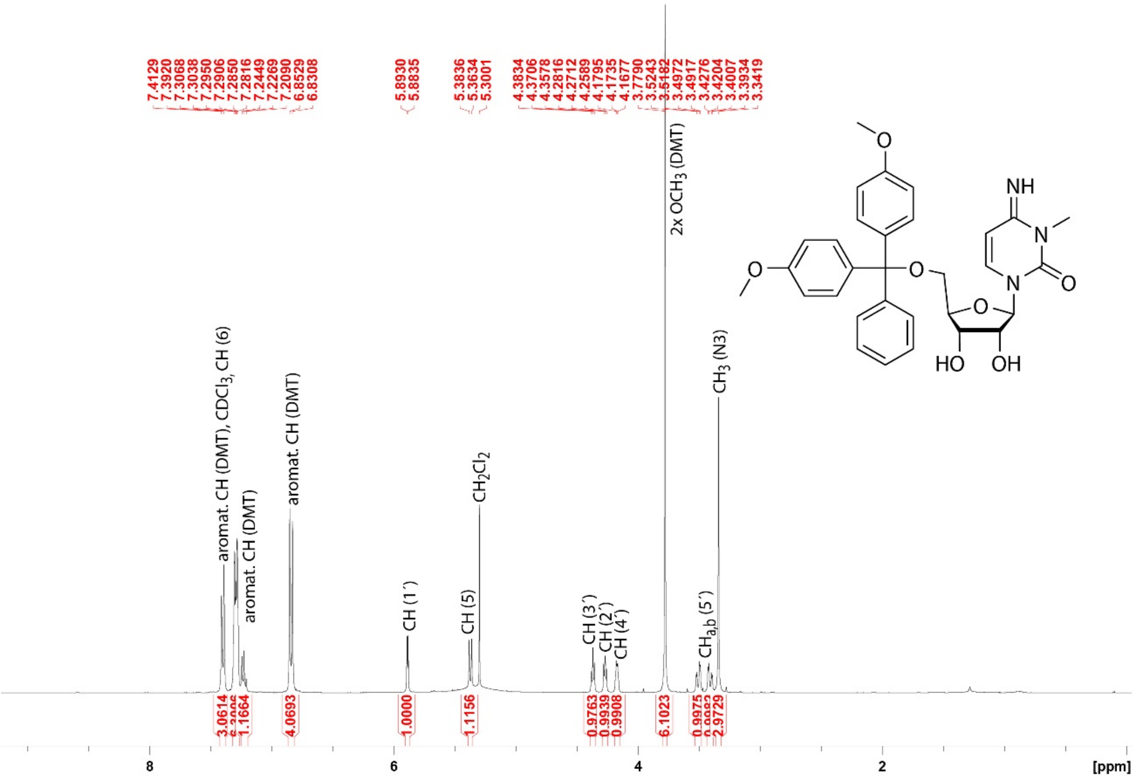

^{13}C-NMR (101 MHz, $CDCl_3$)

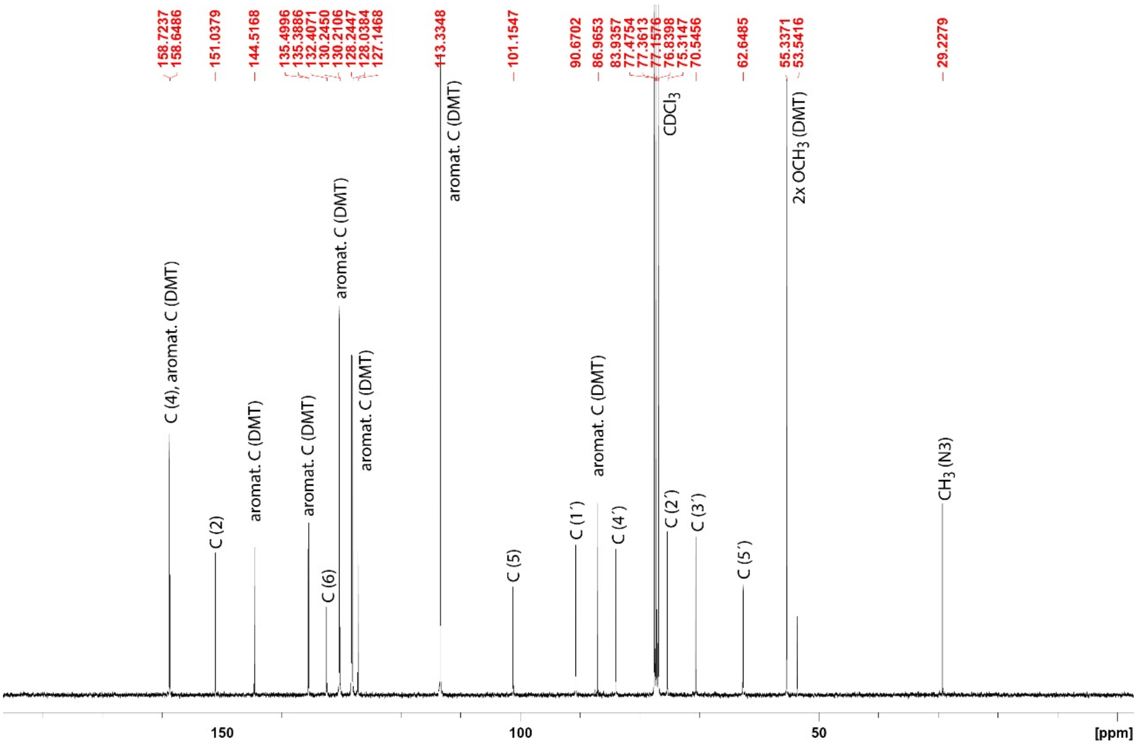

NMR spectra of compound 3

^{1}H-NMR (400 MHz, $CDCl_3$)

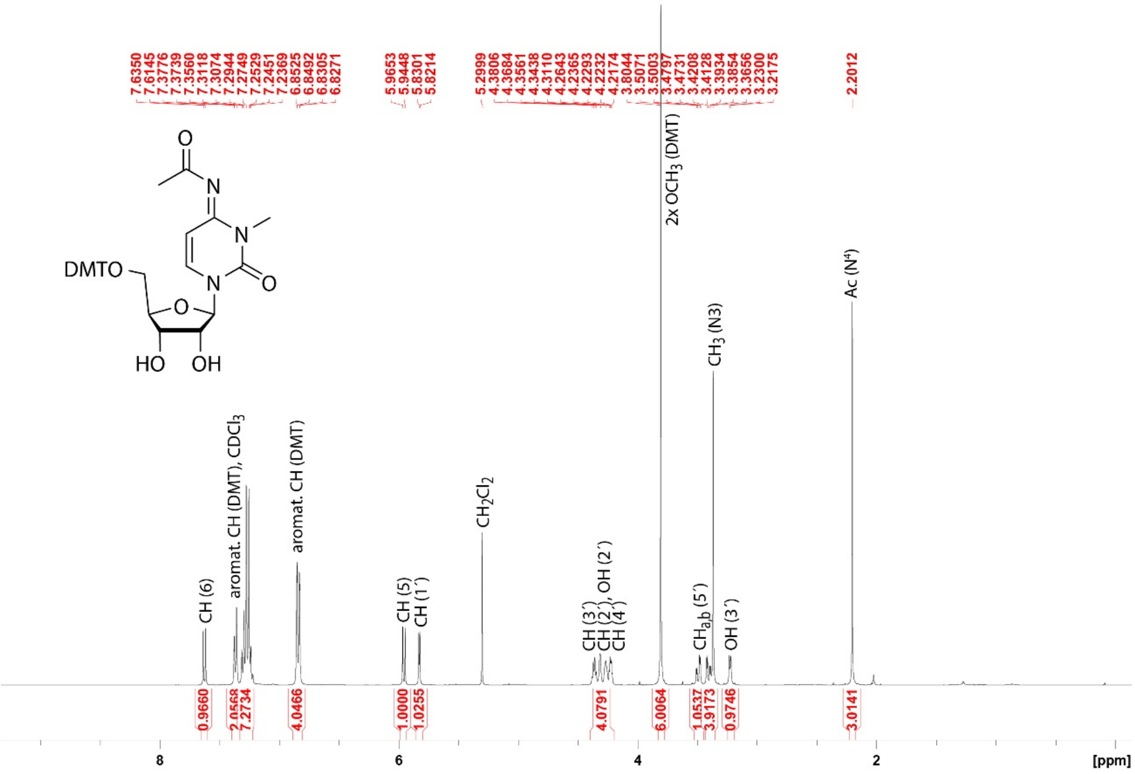

^{13}C-NMR (101 MHz, $CDCl_3$)

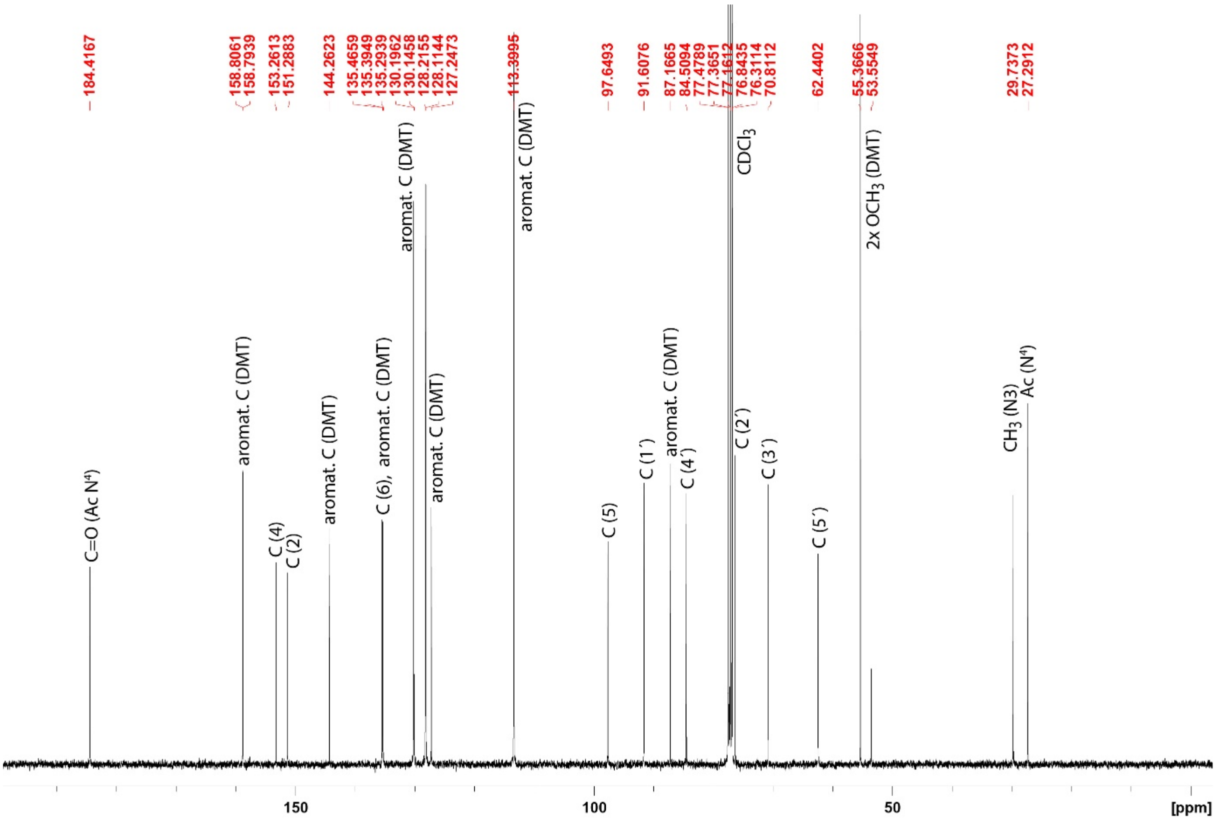

NMR spectra of compound 4a

^{1}H-NMR (400 MHz, d_6-DMSO)

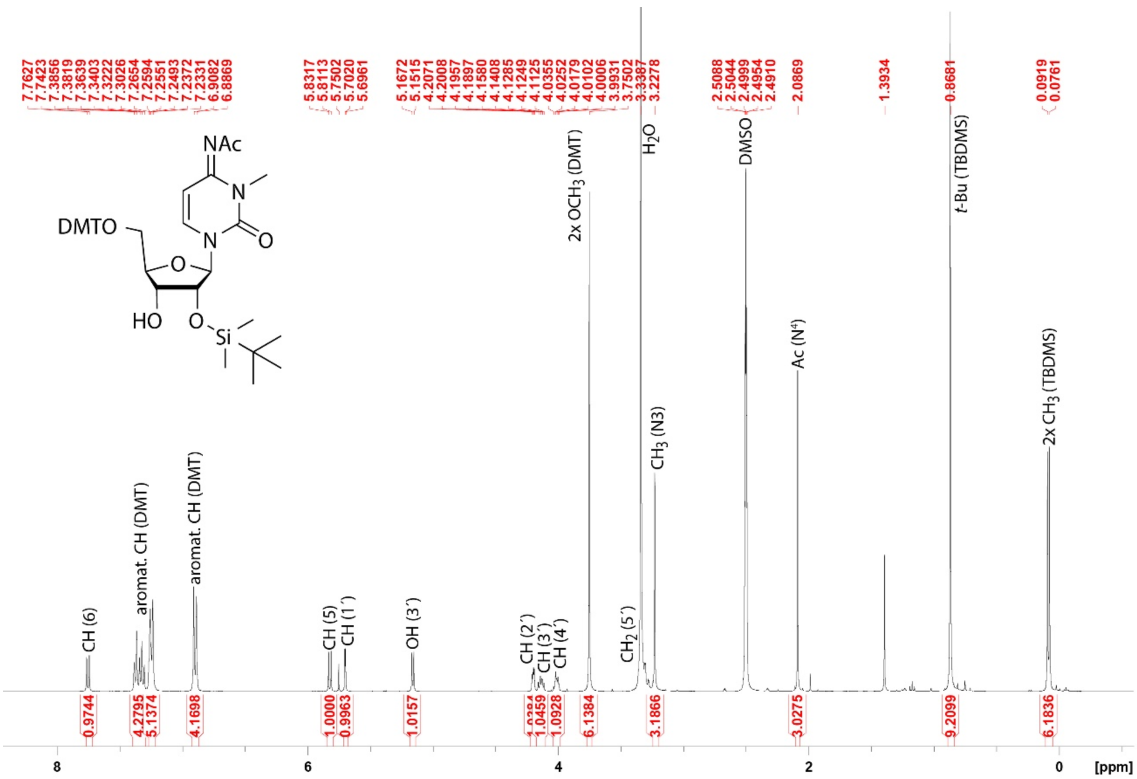

^{13}C-NMR (101 MHz, d_6-DMSO)

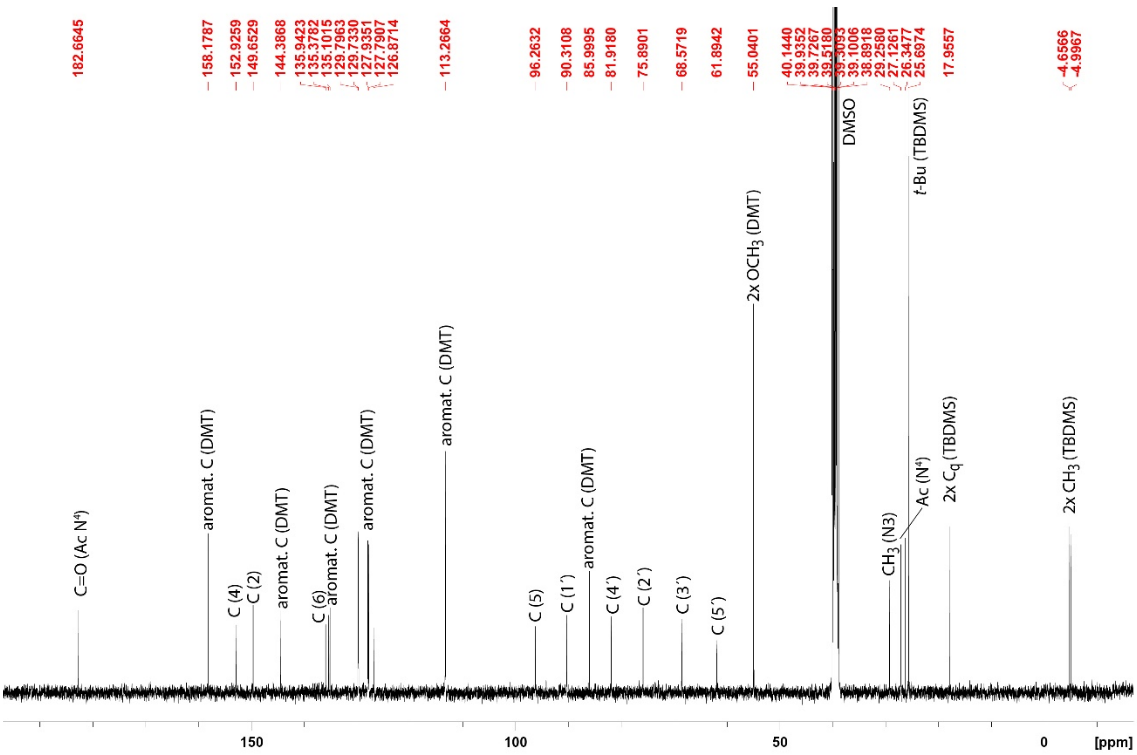

NMR spectra of compound 4b

^{1}H-NMR (400 MHz, d_6-DMSO)

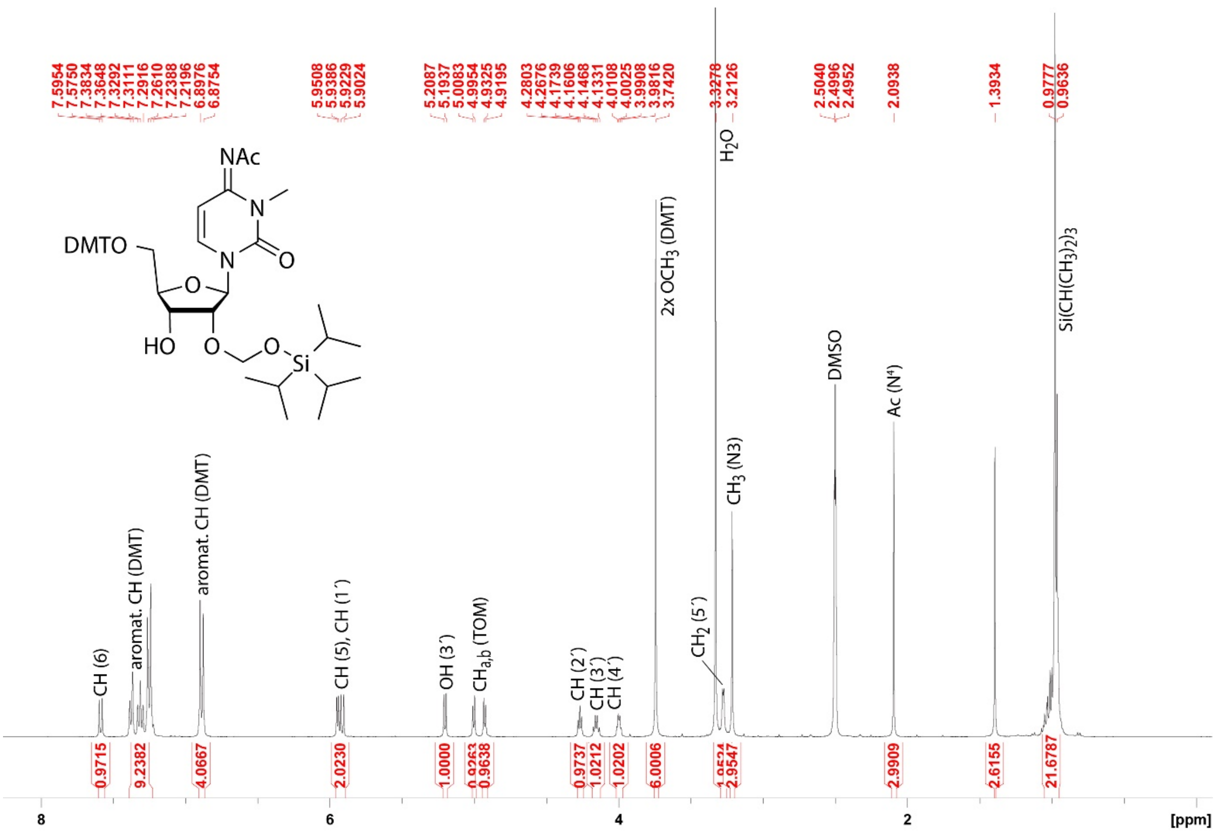

^{13}C-NMR (101 MHz, d_6-DMSO)

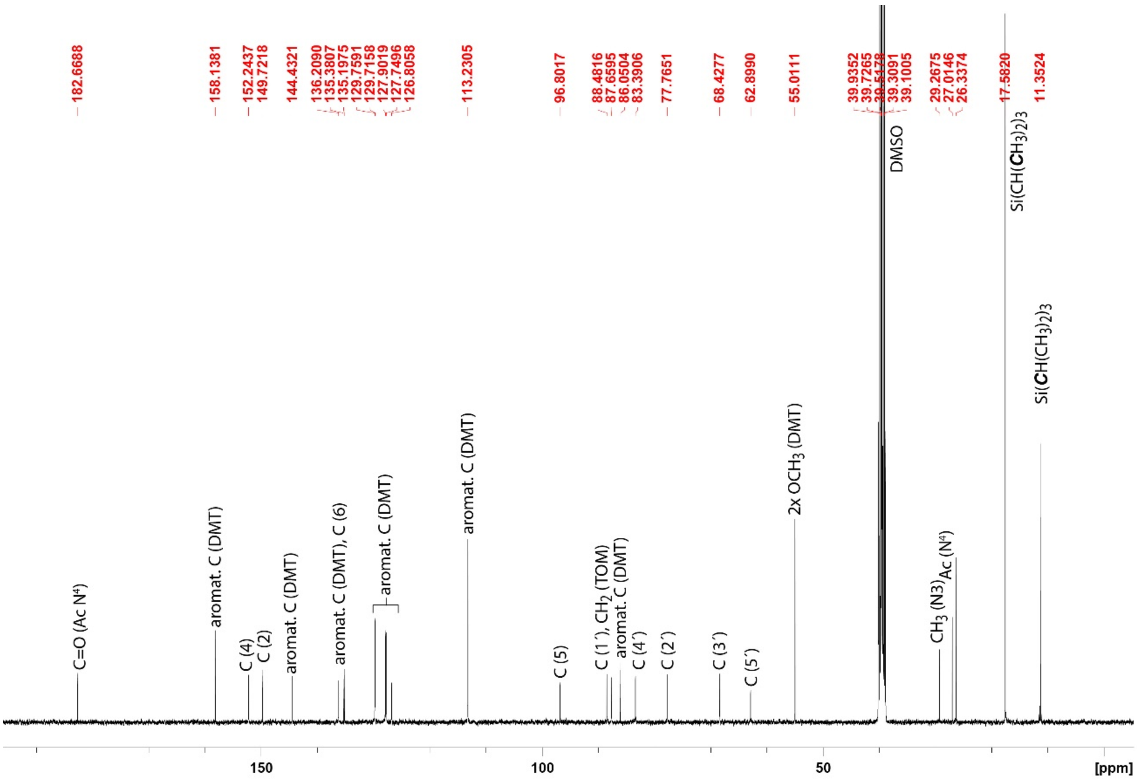

NMR spectra of compound 5a

^{1}H-NMR (400 MHz, $CDCl_3$)

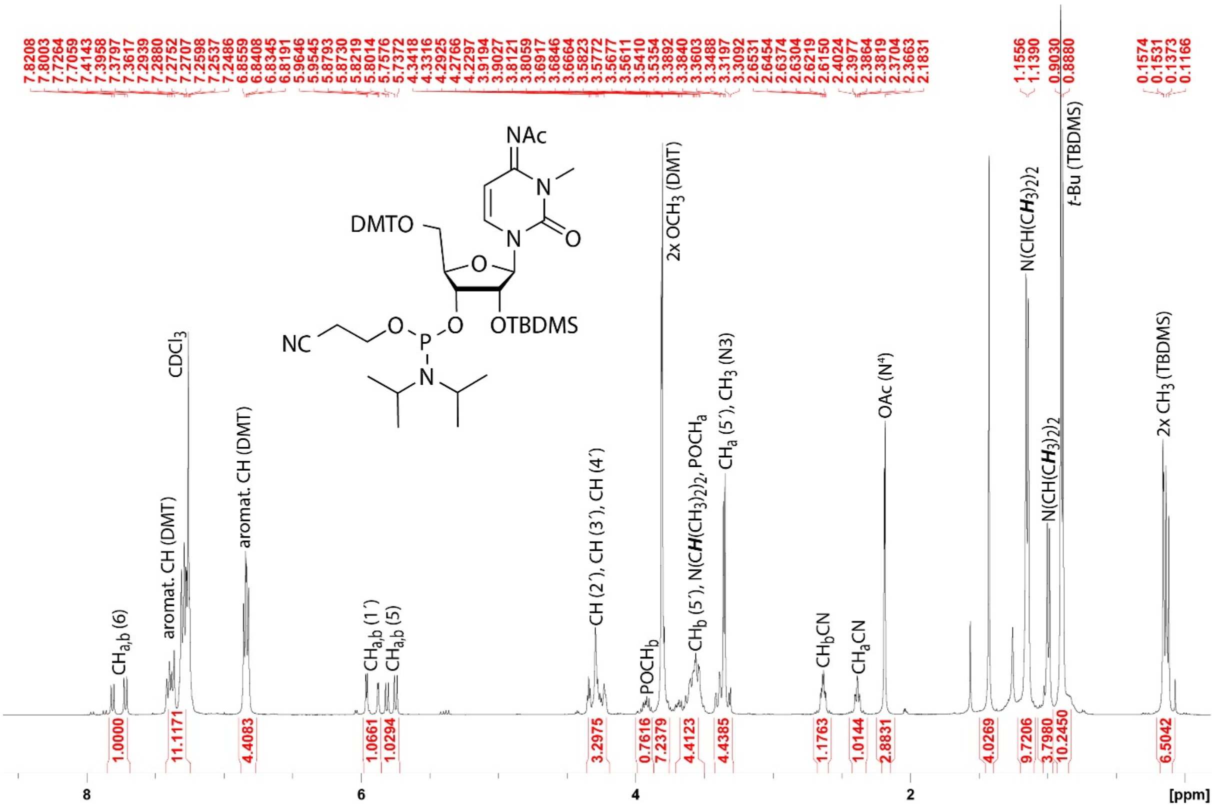

^{13}C-NMR (101 MHz, $CDCl_3$)

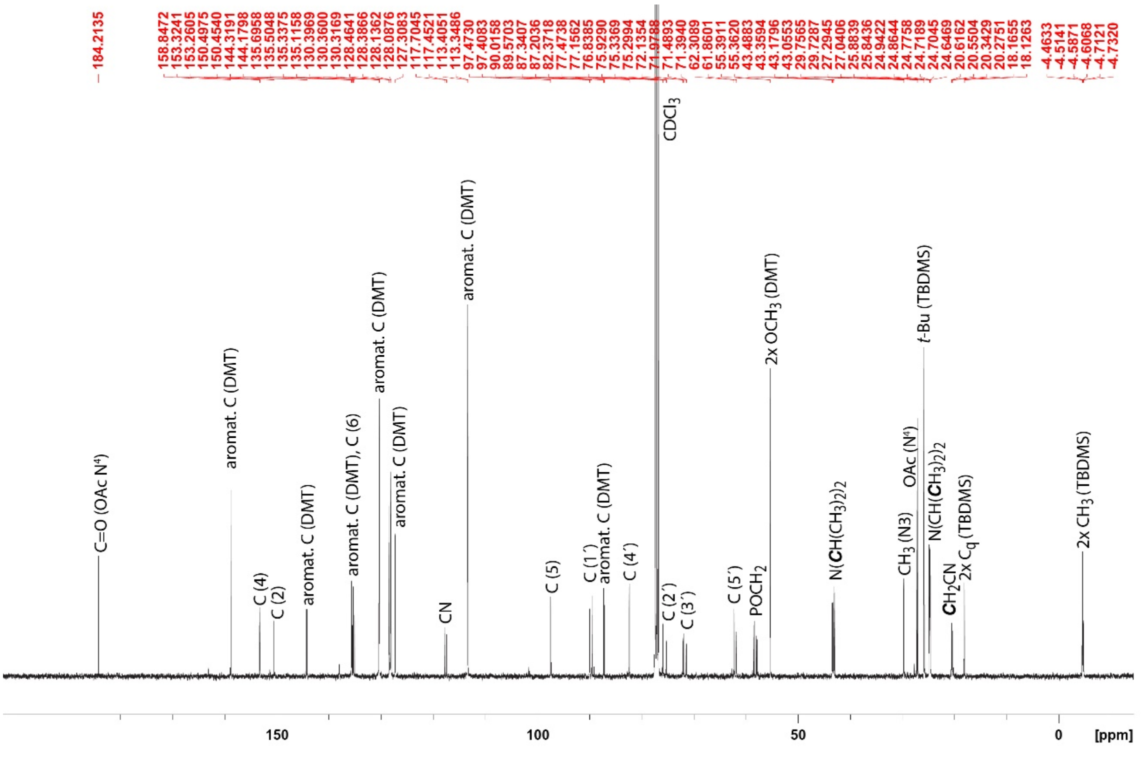

^{31}P-NMR (162 MHz, $CDCl_3$)

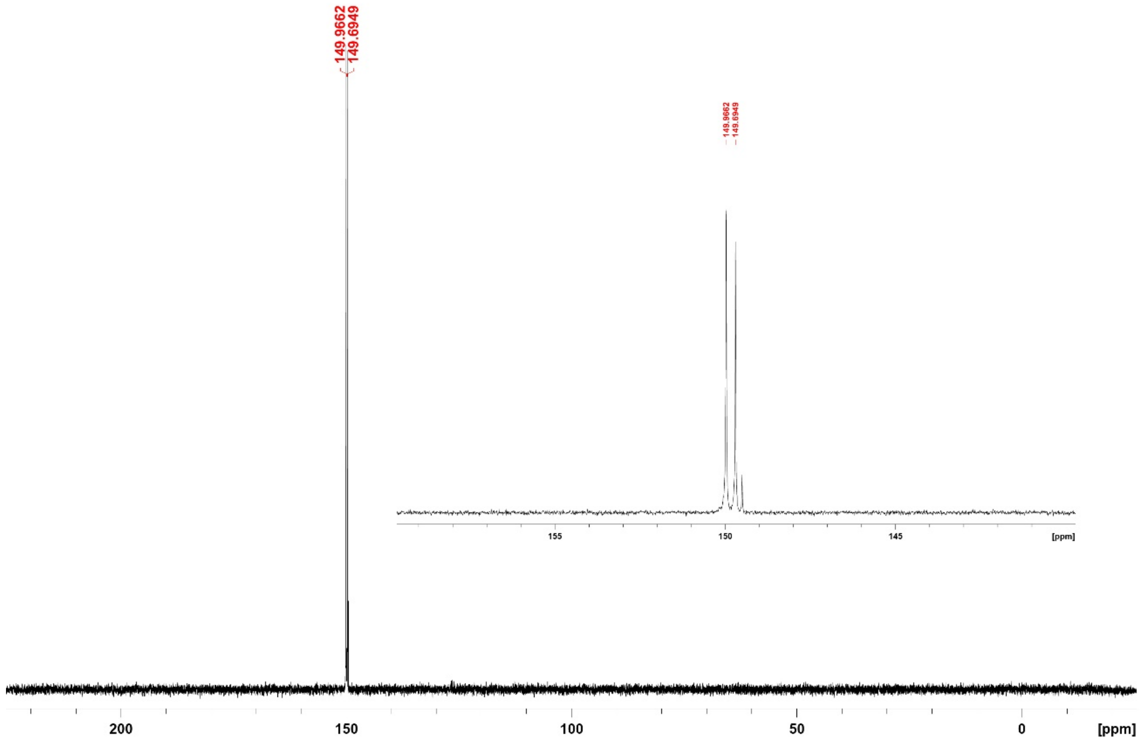

NMR spectra of compound 5b

^{1}H-NMR (400 MHz, $CDCl_3$)

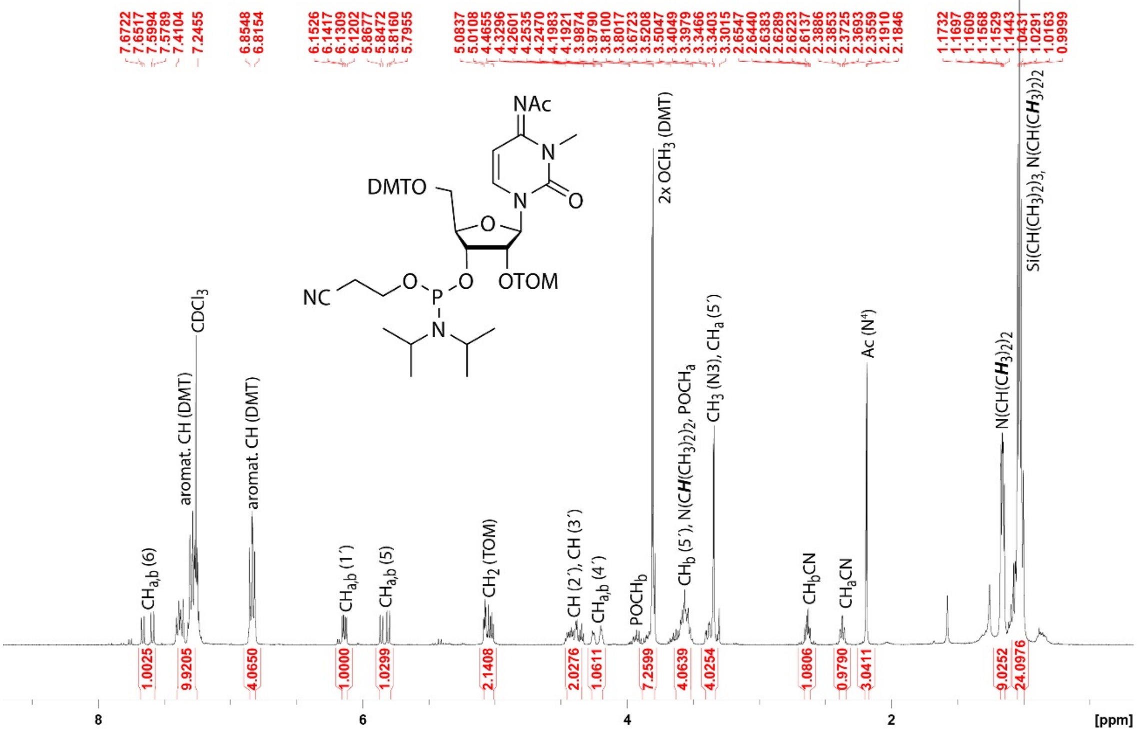

^{13}C-NMR (101 MHz, $CDCl_3$)

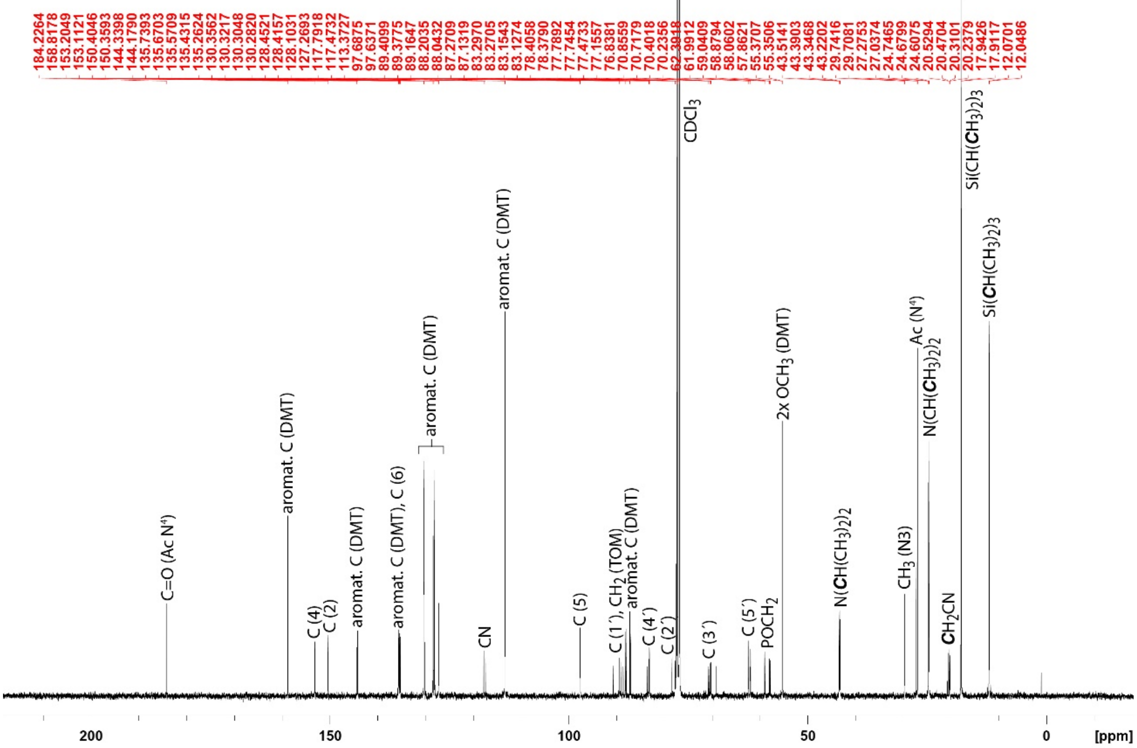

^{31}P-NMR (162 MHz, $CDCl_3$)

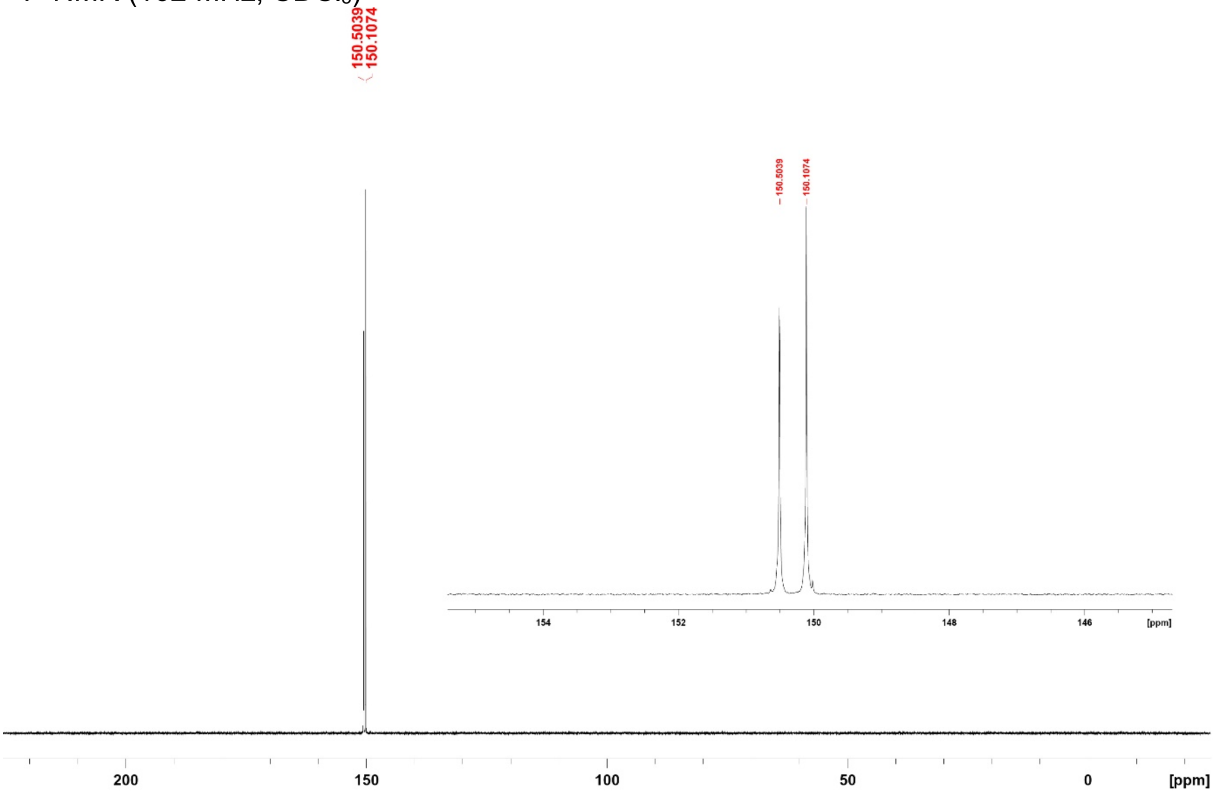

8. RNA solid-phase synthesis of m^3C modified RNA

Standard phosphoramidite chemistry was applied for RNA strand elongation and incorporation of 3-methylcytidine (N^4-Ac, 2'-*O*-TOM **5a** and N^2-Ac, 2'-*O*-TBDMS **5b**; >98% coupling yield). *N*-Acetyl-2'-*O*-TOM protected nucleoside phosphoramidite building blocks and 2'-*O*-Tbs 1000 Å CPG solid support were purchased from ChemGenes, All oligonucleotides were synthesized on an ABI 392 Nucleic Acid Synthesizer following standard methods: detritylation (90 sec) with dichloroacetic acid/1,2-dichloroethane (4/96); coupling (5.0 min) with phosphoramidites/acetonitrile (100 mM, 200 µL) and benzylthiotetrazole / acetonitrile (300 mM, 500 µL); capping (2 x 25 sec) with Cap A/Cap B (1/1) for m^3C modified RNA, Cap A: 4-(dimethylamino)-pyridine/acetonitrile (500 mM), Cap B: acetic anhydride/*sym*-collidine/acetonitrile (2/3/5). Solutions of phosphoramidites, tetrazole and Cap were dried over activated molecular sieves (3 Å) overnight.

9. Deprotection and purification of m^3C modified RNA

For basic deprotection of m^3C modified RNA, the solid support was treated with a mixture of aqueous ammonia (28-30%, 0.50 mL) and ethanol (0.50 mL) for 4 hours at 50 °C. Then, the supernatant was removed and the solid support was washed twice with 0.5 ml ethanol/H_2O (1/1). Combined supernatant and washings were evaporated to dryness and the residue was dissolved in a solution of tetrabutylammonium fluoride in tetrahydrofuran (1.0 M, 1.5 mL) and incubated for 14 hours at 37 °C for removal of 2'-*O*-silyl protecting groups. The reaction was quenched by addition of triethylammonium acetate/H_2O (1.0 M, 1.5 mL, pH 7.4). Tetrahydrofuran was removed under reduced pressure and the sample was desalted with size-exclusion column chromatography (GE Healthcare, HiPrep™ 26/10 Desalting; Sephadex G25) eluting with H_2O; collected fractions were evaporated and the RNA dissolved in H_2O (1 mL). The crude RNA was purified by anion exchange chromatography on a semipreparative Dionex DNAPac® PA-100 column (9 mm x 250 mm) at 80 °C with a flow rate of 1 mL/min (solvent A was 25 mM Tris-HCl (pH 8.0) and 20 mM $NaClO_4$ in 20% aqueous acetonitrile; solvent B was 25 mM Tris-HCl (pH 8.0) and 0.6 M $NaClO_4$ in 20% aqueous acetonitrile; for the 33 nt RNAs depicted in Figure 1 a gradient of 25 – 40 % B in 23 min was used). Fractions containing RNA were diluted with 0.1 M triethylammonium bicarbonate solution, loaded on a C18 SepPak Plus® cartridge (Waters/Millipore), washed with H_2O and eluted with acetonitrile/H_2O (1/1). Crude and purified RNA were analyzed by anion exchange chromatography on a GE Healthcare Äkta Explorer HPLC System containing a Dionex DNAPac® PA-100 column (4 mm x 250 mm) at 80 °C with a flow rate of 1 mL/min; a gradient of 0 – 60 % B (in 47 min) was used; solvent A was 25 mM Tris-HCl (pH 8.0) and 20 mM $NaClO_4$ in 20% aqueous acetonitrile; solvent B was 25 mM Tris-HCl (pH 8.0) and 0.6 M $NaClO_4$ in 20% aqueous acetonitrile. HPLC traces were recorded at UV absorption by 260 nm. RNA quantification was performed on an Implen P300 Nanophotometer.

***Note**: Application of a mixture of aqueous methylamine (40 %, 0.50 mL) and aqueous ammonia (28 %, 0.50 mL) for 12 min at 65°C resulted in formation of m^4m^3C modified RNA (Figure 1B).*

10. Mass spectrometry of oligoribonucleotides

RNA samples (3 µL) were diluted with 40 mM Na_2H_2(EDTA)/H_2O (5/4) for a total volume of 30 µL, injected onto a C18 XBridge column (2.5 µm, 2.1 mm x 50 mm) at a flow rate of 0.1 mL/min and eluted with 0 - 100 % B gradient at 30 °C (Eluent A: 8.6 mM triethylamine, 100 mM 1,1,1,3,3,3-hexafluoroisopropanol in H_2O; Eluent B: methanol). RNA HPLC runs were analyzed on a Finnigan LCQ Advantage Max electrospray ionization mass spectrometer with 4.0 kV spray voltage in negative mode.
